# A rare case of post-traumatic high-flow priapism requiring endovascular salvage with bilateral superselective microcoil embolization

**DOI:** 10.1093/jscr/rjab077

**Published:** 2021-03-08

**Authors:** Aman B Williams, Lauren G Lax

**Affiliations:** Department of Vascular Surgery, Gold Coast University Hospital, Southport 4215, Australia; Intensive Care Unit, Gold Coast University Hospital, Southport 4215, Australia

## Abstract

Post-traumatic high-flow priapism is a rare occurrence, with potentially debilitating long-term erectile dysfunction if left unaddressed. Even rarer, however, is for the priapism symptoms to be caused by a single cavernosal arterial pseudoaneurysm, with feeding vessels from the distal branched vessels of ‘both’ the left and right internal pudendal arteries. To the best of our knowledge, we present the first documented case of endovascular salvage utilizing superselective microcoil embolization in the treatment of high-flow priapism caused by a singular pseudoaneurysm with bilateral inflow. Timing of symptoms, interpretation of imaging, multidisciplinary discussions, procedural risk, arterial anatomy and choice of embolic agent were all careful considerations in this case. Following embolization, this young gentleman ultimately had a successful angiographic result, normalization of his cavernosal artery peak systolic velocity on ultrasound and a full return to normal erectile function by 6 months.

## INTRODUCTION

Non-ischaemic high-flow priapism (HFP) is an uncommon complication of blunt pelvic trauma, which is caused by cavernosal arterial injury and resultant pseudoaneurysm or arterial-lacunar fistula formation [1]. An abundance of oxygenated blood causes a semi-rigid corpora cavernosa, with resulting non-ischaemic erection unrelated to sexual stimulation. Usually painless, HFP carries long-term morbidity if untreated due to chronic corporal fibrosis, tissue necrosis and erectile dysfunction [2].

## CASE REPORT

**Figure 1:**
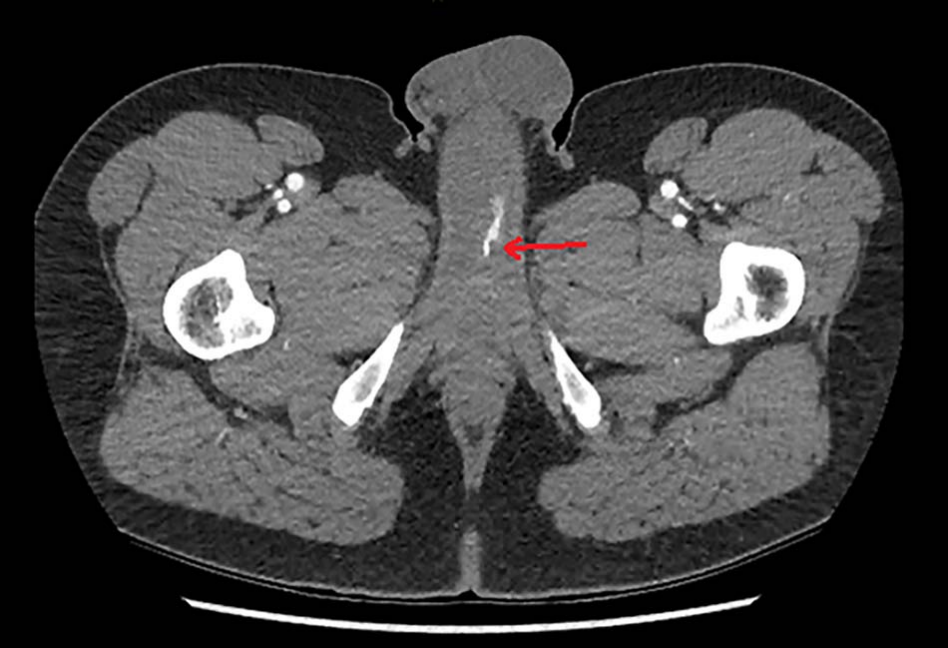
CTA demonstrating an axial view of contrast enhancement within the region of the left cavernosal artery, indicative of an arterial pseudoaneurysm (red arrow).

**Figure 2:**
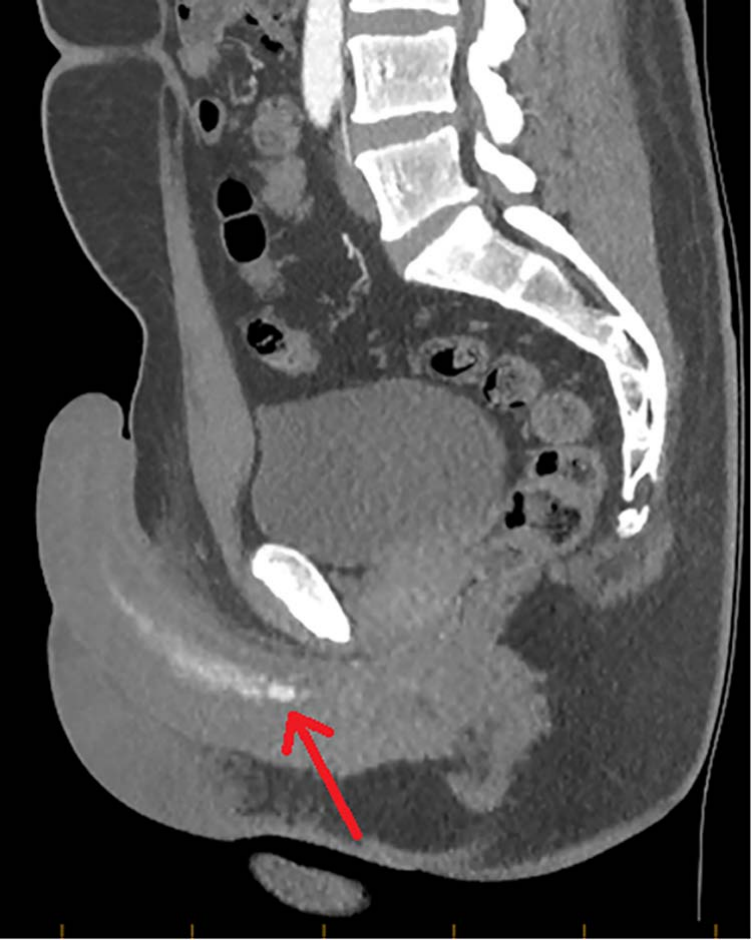
CTA demonstrating a sagittal view of contrast enhancement within the region of the left cavernosal artery and mid-to-distal corpus cavernosum (red arrow).

A 28-year-old gentleman presented with a non-tender, persistently rigid penis 2 weeks after a blunt force injury to his perineum. There was no penetrative damage or other symptoms, with an unremarkable background medical history. Pain-free and well, initial investigation with Doppler ultrasound (US) was sought. US demonstrated some hypoechoic tissue within the corpus cavernosum, but otherwise there was no other soft-tissue abnormality. Colour Doppler did however find an abnormally low peak-systolic-velocity (PSV) in the left cavernosal artery. As such computed tomography angiography (CTA) was arranged, showing an asymmetrical arterial-phase blush within the proximal portion of the left corpus cavernosum, neighbouring the left cavernosal artery (Figs 1 and 2). With no other radiographic genitourinary abnormality, HFP was diagnosed, secondary to arterial pseudoaneurysm.

Following joint trauma–urology–vascular surgery multidisciplinary team meeting, the patient was consulted regarding treatment options, including conservative management, use of a phosphodiesterase-type-5 (PDE5) inhibitor or digital subtraction angiography (DSA) with embolization. After appreciating the risks versus benefits of these options, the patient proceeded to DSA for endovascular interrogation and repair.

**Figure 3:**
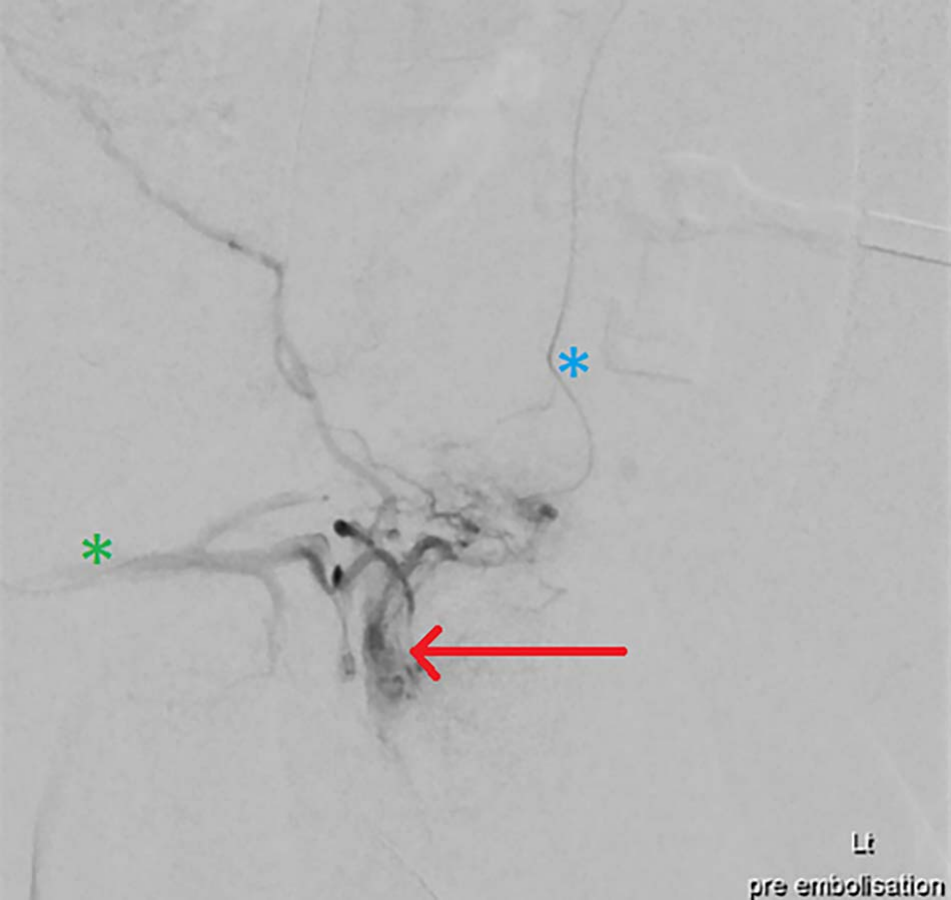
DSA via superselective cannulation of the left (blue asterix) and right (green asterix) distal branches of bilateral internal pudendal arteries; note the arterial pseudoaneurysm (red arrow) pre-embolization.

Under general anaesthetic, US-guided puncture of the left common femoral artery (CFA) was performed, with placement of a long 45cm 5-French Flexor-Sheath (COOK Medical). A 0.035 Glidewire (Terumo) and Rim catheter (Merit Medical) was used to go up and over the aortic bifurcation and to cannulate the right internal iliac artery. The Rim catheter was exchanged for a Van Schie 2 catheter (COOK Medical), with contrast angiography performed from the origin of the right internal pudendal artery (IPA). A pseudoaneurysm was confirmed, arising from the distal branched vessels of the right IPA, notably with contralateral IPA communication. Using a 0.014 wire and a 150 cm Excelsior SL-10 2.4-French microcatheter (Stryker), the pseudoaneurysm was cannulated, with superselective contrast angiography confirming position (Fig. 3).

**Figure 4:**
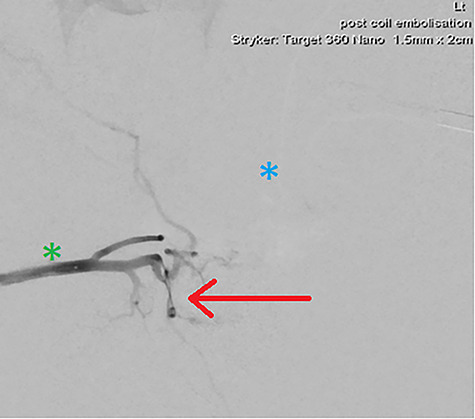
DSA demonstrating significantly reduced flow into the cavernosal artery pseudoaneurysm post-coil embolization (red arrow); note the left (blue asterix) and right (green asterix) distal branches of bilateral internal pudendal arteries for reference.

**Figure 5:**
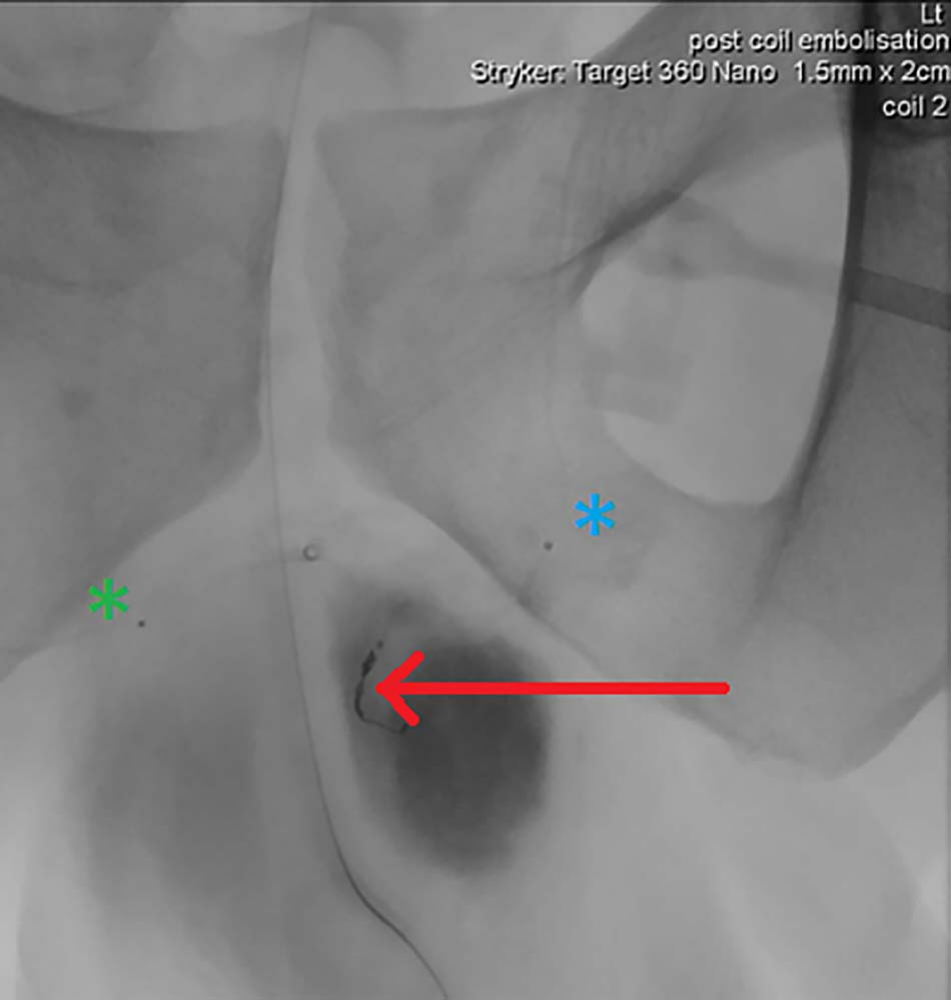
Final intraoperative fluoroscopic image demonstrating microcoil placement (red arrow); note the microcatheter placement within the left (blue asterix) and right (green asterix) distal branches of bilateral internal pudendal arteries for reference

Heparin (3000 IU) was provided. Given contralateral feeding vessels, the right CFA was cannulated with the aforementioned steps mirrored, placing a microcatheter in the pseudoaneurysm via the left IPA. Two Target Neurovascular Microcoils (Stryker) were deployed at the pseudoaneurysm neck, with bilateral microcatheter angiography confirming satisfactory occlusion (Figs 4 and 5). All devices were removed, with Proglide (Abbott) arterial closure.

His recovery was uncomplicated, and a Penile Doppler the next day demonstrated normal cavernosal artery PSV. Follow-up 1 month later yielded significant symptomatic improvement, with completely normal erectile function after 6 months.

## DISCUSSION

Priapism alone is a clinical diagnosis; however, further investigation allows exclusion of ischaemia and planning of early management. With a pain-free patient, penile ischaemia was not a concern in this case, as such corporal blood gas (CBG) was not pursued. CBG is useful in confirming suspicion for ischaemic priapism (e.g. acidosis, hypoxia, hypercapnia and glucopenia), and if deduced, it is a urological emergency.

Though not considered routine, US is useful in differentiating ischemic versus non-ischaemic priapism. In HFP, Doppler of the corpora cavernosa may show the pathognomonic arterial waveform of low resistance and high velocity—carrying nearly 100% sensitivity for HFP [3]. This was not evident in our patient, with instead a contradictory low PSV in the left cavernosal artery—most likely due to the disease being too proximal to image. The region of hypoechoic tissue detected may represent the arteriocavernosal fistula or pseudoaneurysm itself, though more often is the oedematous erectile tissue—hence, prompting CTA for further delineation [4].

In the setting of pelvic trauma, CTA serves several key purposes, including identifying the HFP pathology, excluding other injury and providing relevant arterial mapping to plan a surgical approach. With symptoms of priapism and an ambiguous US, CTA in this case, radiographically diagnosed the presence of a left corpus cavernosum arterial pseudoaneurysm. Though not robustly evidenced in current literature, a feature suggestive of HFP on CTA is a jetted-flow of arterial blood within the corpus cavernosum and associated arterial pooling in its bulbar segments [5].

In the short term, ischaemic sequalae from HFP is rare; hence, brief delays to plan intervention are acceptable. Conservative management with bed rest and icepacks coupled with clinical surveillance is first-line; however, given the fortnight of priapism symptoms’ pre-presentation in our patient, multidisciplinary consensus was that conservative management would be insufficient. Thus, endovascular salvage was pursued. Despite DSA’s high diagnostic and therapeutic yields, the risk of long-term erectile dysfunction needs to be clearly disclosed, bearing a 5–39% risk [6]. Though rare, other risks include purulent cavernositis, penile gangrene, gluteal ischaemia and a 30% recurrence rate [6].

To our knowledge, this is the first case that describes post-traumatic HFP with bilateral feeding vessels into a single arterial pseudoaneurysm. Bilateral arterial pseudoaneurysms have been reported; however, they clearly arise from their relevant ipsilateral distal internal iliac artery branches without communication. Given the miniscule size of these distal vessels and long tortuous catheters in IPAs bilaterally, heparinization is essential to prevent needless vessel thrombosis. In cases of bilateral arterial pseudoaneurysm formation, there has been variable success reported regarding unilateral CFA versus bilateral CFA embolization access in the resolution of HFP symptoms. In our patient, the decision for bilateral feeding vessel embolization was made, given the lacklustre of current evidence for successful unilateral therapy, with several reports of patients having to undergo repeat contralateral access embolization procedures [7].

Current choices of embolic agent for HFP can include coils, ethanol, sclerosant, gelatine and glue. Microcoil deployment carries several advantages, especially its radiopaque nature, rapid accurate deployment and cavity packability, i.e. into a pseudoaneurysm. This allows treatment of the culprit lesion, without inadvertent obliteration of neighbouring tributaries. Furthermore, we found coil placement served (opportunistically) as a marker for targeted perineal-US locating the pseudoaneurysm without any compromise to the image quality in this case. Though a good result was achieved with coils, liquid embolic agents should be considered when faced with multiple unilateral feeding vessels to promote eventual recanalization of native arterial supply and to reduce the risk of future erectile dysfunction [8].
